# Deep learning-based osteochondritis dissecans detection in ultrasound images with humeral capitellum localization

**DOI:** 10.1007/s11548-023-03040-8

**Published:** 2024-01-17

**Authors:** Kenta Sasaki, Daisuke Fujita, Kenta Takatsuji, Yoshihiro Kotoura, Masataka Minami, Yusuke Kobayashi, Tsuyoshi Sukenari, Yoshikazu Kida, Kenji Takahashi, Syoji Kobashi

**Affiliations:** 1 Graduate School of Engineering, University of Hyogo, Himeji, Hyogo Japan; 2 Department of Orthopaedics, Graduate School of Medical Science, Kyoto Prefectural University of Medicine, Kyoto, Japan

**Keywords:** Osteochondritis dissecans, Humeral capitellum, Ultrasound images, Deep learning

## Abstract

**Purpose:**

Osteochondritis dissecans (OCD) of the humeral capitellum is a common cause of elbow disorders, particularly among young throwing athletes. Conservative treatment is the preferred treatment for managing OCD, and early intervention significantly influences the possibility of complete disease resolution. The purpose of this study is to develop a deep learning-based classification model in ultrasound images for computer-aided diagnosis.

**Methods:**

This paper proposes a deep learning-based OCD classification method in ultrasound images. The proposed method first detects the humeral capitellum detection using YOLO and then estimates the OCD probability of the detected region probability using VGG16. We hypothesis that the performance will be improved by eliminating unnecessary regions. To validate the performance of the proposed method, it was applied to 158 subjects (OCD: 67, Normal: 91) using five-fold-cross-validation.

**Results:**

The study demonstrated that the humeral capitellum detection achieved a mean average precision (mAP) of over 0.95, while OCD probability estimation achieved an average accuracy of 0.890, precision of 0.888, recall of 0.927, F1 score of 0.894, and an area under the curve (AUC) of 0.962. On the other hand, when the classification model was constructed for the entire image, accuracy, precision, recall, F1 score, and AUC were 0.806, 0.806, 0.932, 0.843, and 0.928, respectively. The findings suggest the high-performance potential of the proposed model for OCD classification in ultrasonic images.

**Conclusion:**

This paper introduces a deep learning-based OCD classification method. The experimental results emphasize the effectiveness of focusing on the humeral capitellum for OCD classification in ultrasound images. Future work should involve evaluating the effectiveness of employing the proposed method by physicians during medical check-ups for OCD.

## Introduction

Osteochondritis dissecans (OCD) of the humeral capitellum is a common cause of elbow disorders among young throwing athletes. The progression of OCD can be divided into three stages: the radiolucent stage, the fragmentation stage, and the loose body stage [1]. In the radiolucent stage, the lesions start in the subchondral bone with intraosseous subchondral osteopenia and present as translucency on X-ray. As the disease progresses, a sclerotic ring can be seen that distinguishes the lesion from the surrounding healthy bone, and the lesion is separated from the surrounding tissue as a fragment. As the disease becomes worse, the lesion has completely separated from the surrounding tissue, forming a loose body within the joint. Symptoms of OCD vary depending on the stage of the disease. In the radiolucent stage, few people feel any pain and no symptoms are present. In the fragmentation stage, the elbow may become painful, and the range of motion of the elbow becomes restricted. In the loose body stage, the pain becomes more severe, and irreversible bone deformity may occur [2–4]. Various studies have shown that OCD of the humeral capitellum may occur in approximately 3% of adolescent baseball players [5–9].

The treatment of OCD typically involves conservative treatment, but sometimes surgery is necessary. Conservative treatment needs a prolonged absence from sports activity. Activity restriction would have a role in stabilizing the OCD lesion and promoting ossification of the lesion. If successful, the bone will be repaired in about one year and the child will be able to play baseball again. Matsuura et al. [4] reported that osteochondrosis of the humeral capitellum can be successfully treated conservatively if treatment is begun in an early stage of the disease. When 84 patients with the early stage OCD (the radiolucent stage) stopped pitching and underwent conservative treatment, OCD lesions of 76 patients (90.4%) healed, on the other hand, only 4 of 33 patients (12.1%) who did not follow the instruction to stop pitching healed. In the case of 17 patients who were diagnosed as the advanced stage (the fragmentation stage), 9 (52.9%) patients who underwent conservative treatment healed, while none of the 23 patient’s OCD lesions who did not follow the instructions healed. As can be seen from this result, 90% of the patients in the radiolucent stage can be successfully treated conservatively, whereas only about half of the patients in the fragmentation stage had effective results after conservative treatment, indicating that early detection in the early stage (the radiolucent stage) is important for healing of the humeral capitellum OCD in growing baseball players. Surgery is generally required in the fragmentation stage and the loose body stage, and mainly consists of removal of the loose body, resection of the deformed bone, and osteochondral grafting [1]. However, it seems difficult to heal the joint deformity in an advanced stage, so early detection and treatment are important.

Current diagnostic methods for OCD are X-ray, computed tomography (CT), magnetic resonance imaging (MRI), and ultrasonography. Among these, one study reported that CT is the best method of examination for OCD [10]. This is because loose bodies of OCD lesions are often missed on radiographs and MRI scans [10]; whereas, CT can accurately determine the location of the lesion as well as the extent of the lesion [4, 11, 12]. On the other hand, ultrasonography is used as a medical screening because many players do not visit a hospital while they are able to continue competing despite the pain. They visit an outpatient clinic only after their condition has become severe, and medical screening using ultrasonography is needed [7]. Ultrasonography is suitable for screening because it is noninvasive, with no risk of radiation exposure, and can detect subtle changes in subchondral bone in the early stages. Not only have some studies [13] shown the usefulness of diagnosing the presence or absence of OCD from ultrasound images, but some have reported better diagnostic performance than MRI [14]. Therefore, screening for OCD by ultrasonography enables early detection and conservative treatment [7, 8, 15–18]. In this screening, orthopedic surgeons and sonographers examine baseball player’s elbows using ultrasound. However, medical check-ups for OCD are conducted only a few times a year, and there is a limited number of specialists available to perform these examinations. Furthermore, it is sometimes difficult to detect OCD lesions and the diagnosis may differ among examiners.

A related study of computer-aided diagnosis of ultrasound images that do not use deep learning is that of Acharya et al. [19]. In this study, the classification of the normal thyroid gland and Hashimoto’s thyroid gland was performed by extracting grayscale features of thyroid ultrasound images using wavelet transform, SVM, decision trees, etc. The SVM achieved the accuracy rate of 82.7%, the sensitivity rate of 93.1%, and the specificity of 69.6%. A study by Fujioka et al. [20] is a study using deep learning. This study compared the accuracy of the convolutional neural network (CNN) model, which uses the GoogLeNet model, with that of three radiologists in classifying benign and malignant breast masses based on ultrasound images. The results showed that the CNN model achieved an area under the curve (AUC) of 0.913, which was equivalent or superior to the radiologists’ accuracy.

Shinohara et al. proposed a detection model using deep learning for OCD of the humeral capitellum which has been demonstrated [21]. It evaluates the entire image and classifies into OCD or normal. The classification model was constructed by transfer learning of three models, ResNet50, MobileNet_v2, and EfficientNet, on images of 40 subjects, and achieved the accuracy rate of 0.818, 0.841, and 0.872, respectively. In addition, object detection of OCD lesion location is performed using YOLOv2. The mAP, which indicates the percentage of matching with the bounding box of the true value, achieved 0.83.

The purpose of this study is to propose a deep learning-based OCD detection method that automatically determines the presence or absence of OCD in ultrasound images. The detection method aims to support medical professionals without specialized expertise to screen patients for OCD using ultrasound images, solving the problem of OCD screening and increasing the possibility of early detection of OCD. The proposed method first detects the humeral capitellum of the elbow, a common site of OCD, by using the YOLO object detection method, and then classifies the detected bounding box into OCD or normal by using VGG16. We hypothesize that restricting the region of interest (ROI) on the humeral capitellum will improve the accuracy of predicting OCD compared to models built using the entire ultrasound image captured by ultrasonographic devices.

### Subjects and materials

**Fig. 1:**
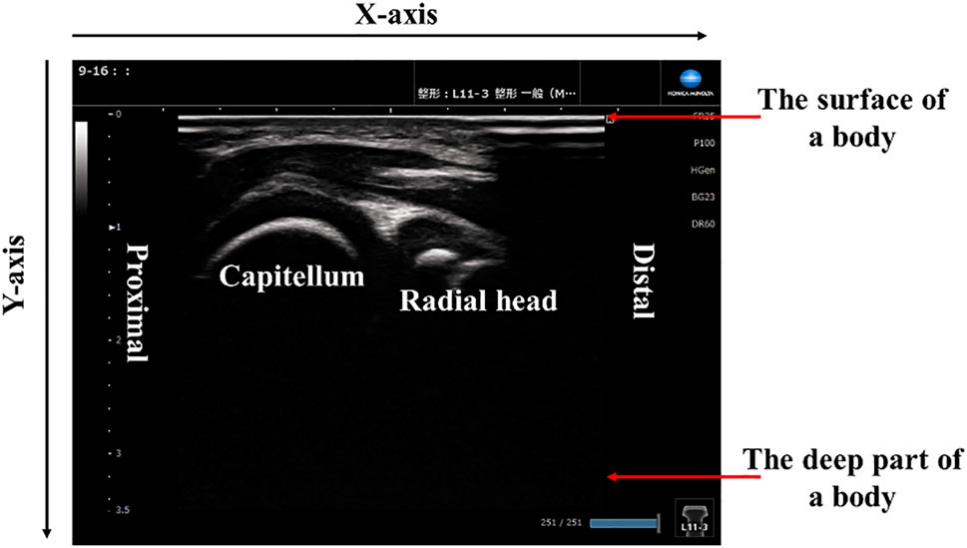
Ultrasound images of the elbow in lateral, posterior, and long-axis imaging directions

The ultrasound images used in this experiment were taken by physicians experienced in medical check-ups of OCD using the five ultrasound systems shown in Table 1. The ultrasound images of the elbow were taken in the lateral posterior long-axis directions. Figure 1 shows ultrasound images of the elbow, where the humeral capitellum of the elbow is located on the x-axis and y-axis. The upper side of the image represents the surface of a body; while, the lower side represents the deeper part of the body (Table 2).

**Table 1:** Ultrasonographic devices

Name of product	Probe Types	Manufacturer
HI VISION Avius	14 or 15 MHz high-frequency Linear probe	Hitachi Medical Corp
Noblus	14 MHz high-frequency Linear probe	Hitachi Medical Corp
ACUSON P300	12 MHz high-frequency Linear probe	Siemens medical solutions
ARIETTA Prologue	15 MHz high-frequency Linear probe	Hitachi Medical Corp
SNiBLE2	15MHz high-frequency Linear probe	KONICA MINOLTA

**Table 2:** Number of subjects used in the experiment

	Number of subjects	Age (average ± standard deviation)	Number of images per subject
OCD	91	11.7 ± 1.9 years old	1–48
Normal	67	10.2 ± 1.3 years old	1–80

Subjects used in this study were all male, 67 with OCD (11.7 ± 1.9 years old) and 91 with normal (10.2 ± 1.3 years old). The OCD diagnosis was confirmed by senior orthopedic surgeons using radiographs of the elbow in 45° flexion in the frontal plane. Although there were various stages of OCD cases, all were regarded as the same OCD group. Two types of data were used in the experiment: movies and still images. Movies were archived in avi format, and divided into frames, which were then used as still images in bmp format. We analyzed only images that include the humeral capitellum, a common site of OCD, but excluded images of the same location, leaving only one image. Still images were also stored as bmp files, and only images including the humeral capitellum were used. Therefore, the number of images varied from subject to subject, with a minimum of 1 and a maximum of 80 images for normal subjects, and a minimum of 1 and a maximum of 48 images for OCD subjects. The size of the images varied from subject to subject, ranging from 400 to 500 pixels in both height and width. This study was conducted with the approval of the ethics committees of the Department of Orthopaedics Graduate School of Medical Science Kyoto Prefectural University of Medicine and the Graduate School of Engineering, University of Hyogo.

## Method

### Overview

In actual practice, physicians diagnose OCD by checking the humeral capitellum of the elbow in ultrasound images shown in Fig. 1. The proposed method follows the physician’s procedure and automatically focuses the ROI on the humeral capitellum in the image. We hypothesize that this improves classification accuracy by eliminating unnecessary information.

**Fig. 2:**
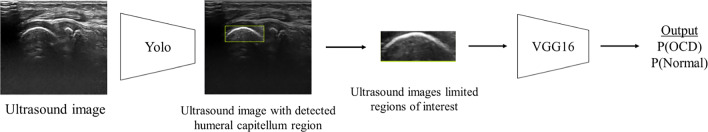
Overview of the proposed method

The proposed method can be divided into two major steps. The first step detects the subchondral bone surface of the humeral capitellum from the ultrasound image using YOLO object detection. The detected bounding box, which encompasses the subchondral bone surface of the humeral capitellum, serves as ROI. The second step estimates the probability of OCD from the ROI using fine-tuned VGG16. Figure 2 shows an overview of the proposed method.

### Step 1. Humeral capitellum detection by YOLO

**Fig. 3:**
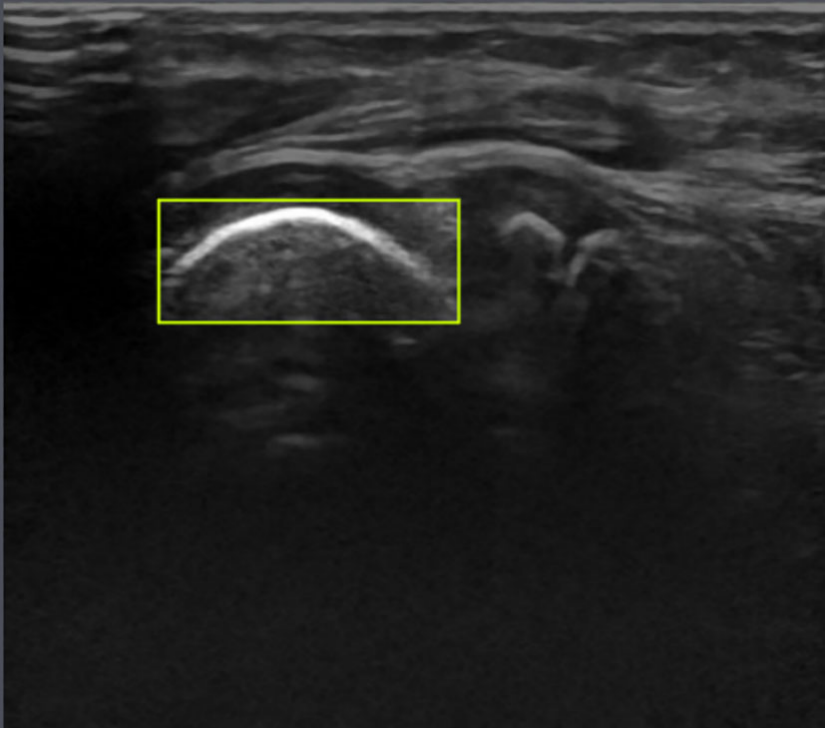
Annotated image by Roboflow

Object detection using YOLO is applied to detect the humeral capitellum in the ultrasound images. YOLOv5s [22], which has been previously trained on the COCO dataset, is used. A model for detecting the humeral capitellum in ultrasound images of the elbow is constructed by transfer learning. Annotation is performed using an annotation tool (Roboflow [23]), with bounding boxes set to encompass the surface of the humeral capitellum in the image (Fig. 3). An image cropped by the predicted bounding box is evaluated as ROI by the second step.

### Step 2. OCD probability estimation using VGG16

**Fig. 4:**
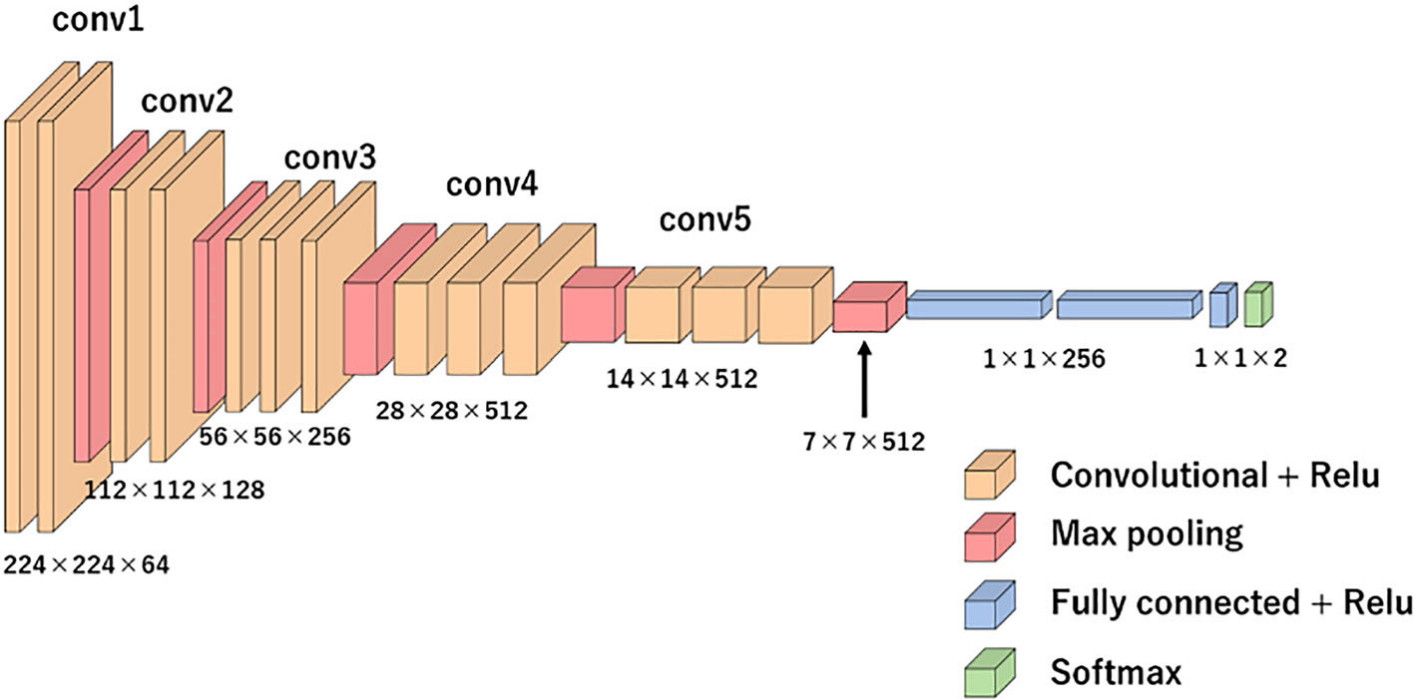
OCD classification model based on VGG16

**Fig. 5:**
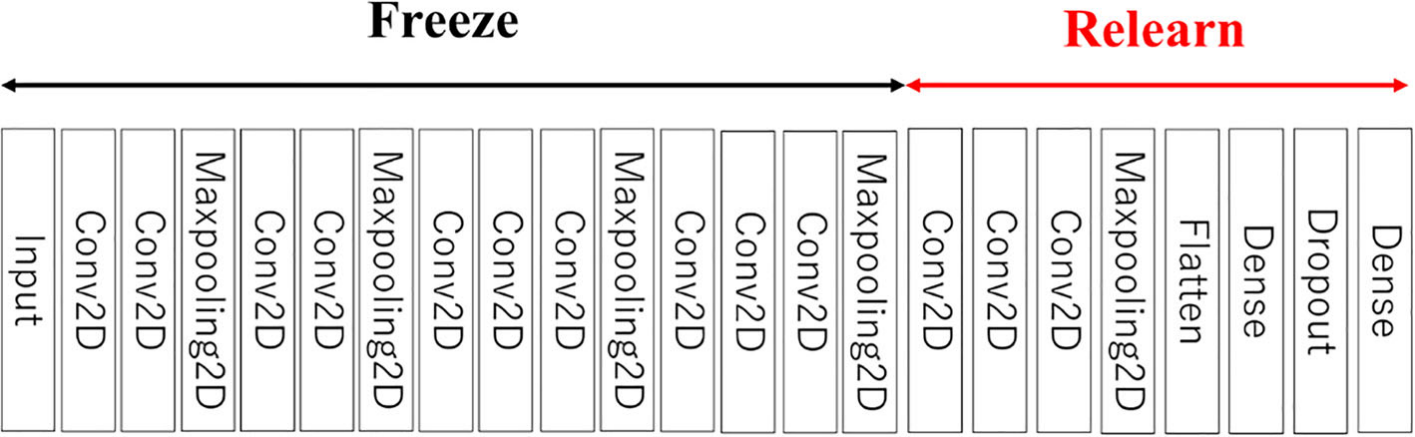
Structure of VGG16 in the fine-tuning of this study

The proposed method estimates OCD probability by classifying the ROI into two classes, OCD or normal, using the VGG16 convolutional neural network structure [24]. The VGG16 model was pre-trained on ImageNet [25] and outputs the probability of each of the 1000 classes for each image. To estimate the probability of OCD, the output layer is replaced with a layer that outputs OCD and normal probabilities. The input size is 224 × 224 and the network structure includes five blocks of two convolutional layers and max pooling. All coupling layers are modified, and a dropout layer with a dropout rate of 0.5 is added. The convolutional layer closest to the output is re-trained with ultrasound images using fine-tuning. Figure 4 shows the network structure used, and Fig. 5 shows the fine-tuning process.

## Results

### Results of the humeral capitellum detection using YOLO

The proposed method was evaluated using ultrasonic images of 158 subjects (67 with OCD and 91 with normal). In this experiment, images whose IoU exceed the threshold at step 1 are only evaluated. The training conditions of YOLOv5s were, 100 as the number of epochs, 128 as the batch size, stochastic gradient descent (SGD) as the optimization function, and 0.01 as the learning rate. Subject based five-fold cross-validation was applied, where the subjects were divided into three sets at a ratio of 3 for training data, 1 for validation data, and 1 for test data, with no overlap between the three sets. The ratio of the number of images between OCD and normal subjects was balanced across folds. Mean average precision (mAP) was used to evaluate the accuracy of YOLO’s humeral capitellum detection, which is calculated using precision and recall defined below.

1 $$\mathrm{Precision}=\frac{\mathrm{TP}}{\mathrm{TP}+\mathrm{FP}}$$

2 $$\mathrm{Recall}=\frac{\mathrm{TP}}{\mathrm{TP}+\mathrm{FN}}$$

where TP represents the number of true positives, FP represents the number of false positives, and FN represents the number of false negatives. TP, FP, and FN are determined using intersection over union (IoU), which measures the overlap between the bounding box of the ground truth and prediction. When the IoU is equal to or higher than a certain threshold, it is considered TP. In this experiment, mAP70 was calculated with an IoU threshold of 0.7.

**Fig. 6:**
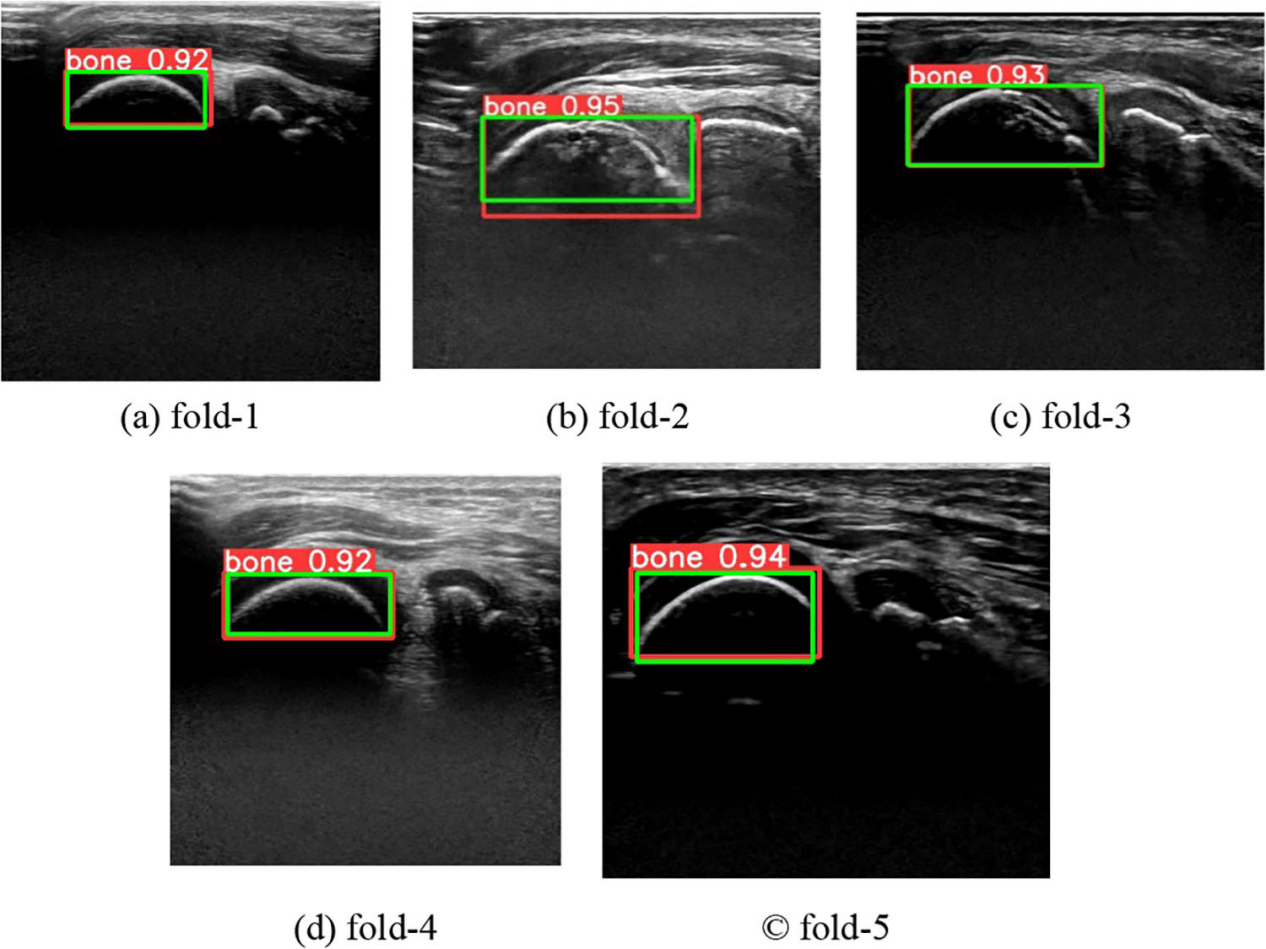
Examples of successful detection in each fold (Green: ground truth, Red: prediction). Numbers show the confidence values

**Fig. 7:**
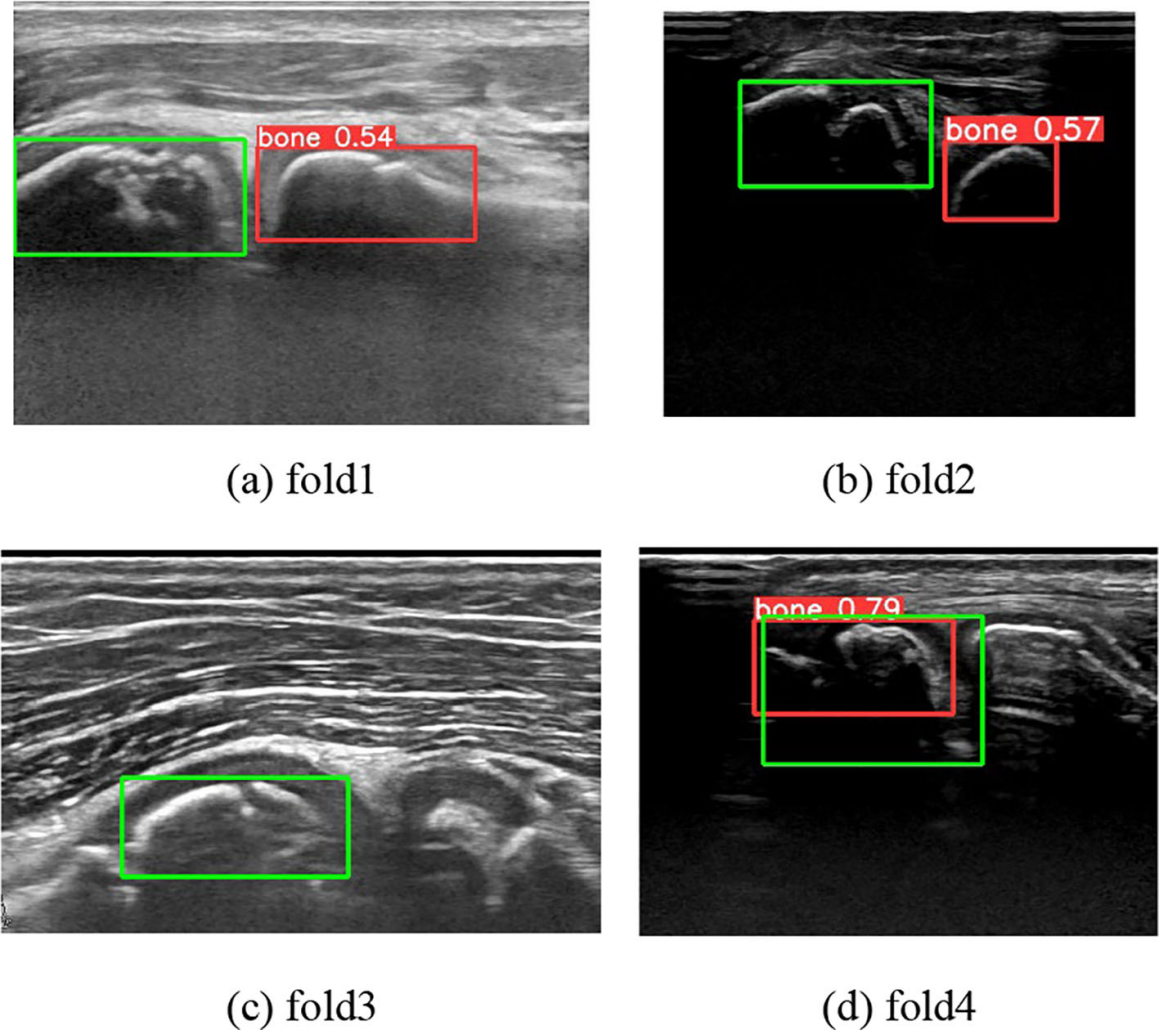
Examples of detection failure in each fold (Green: ground truth, Red: prediction). Numbers show the confidence values

Table 3 shows the mAP70 and the number of images where the humeral capitellum was not detected for each fold. Meanwhile, Figs. 6 and 7 illustrate successful and failed detection examples, respectively. In Fig. 7, different bones were detected in (a) and (b), no bone was detected in (c), and the humeral capitellum was detected but with an IoU less than 0.7 in (d). Out of the 23 failed detections, 18 were from OCD subjects, a possibility due to the irregular and painful bony surface of the OCD humeral in severe cases. The failed images did not proceed to the next step. However, since ultrasonic images are acquired with varying positions and directions, the proposed method can detect the humeral capitellum accurately in most images. Moreover, physicians can easily identify severe OCD cases, so the proposed method remains effective even if some frames fail to detect the humeral capitellum.

**Table 3:** Results of humeral capitellum detection

Fold	1	2	3	4	5
mAP70	0.976	0.970	0.953	0.970	1.000
Number of detection failures (number of subjects)	7/397 (2/33)	7/291 (4/31)	9/230 (4/31)	3/119 (2/32)	0/240 (0/31)

### Results of OCD classification using VGG16

Step 2 of the proposed method was applied to the ultrasound images of 158 subjects (67 with OCD and 91 with normal) in which the humeral capitellum was successfully detected in step 1. The training conditions of VGG16 were set to 50 epochs, 256 batch sizes, SGD as the optimization function, binary_crossentropy as the loss function, and 0.001 as the learning rate. Five-fold cross-validation was applied, and the data were classified in the following proportions: 3 for training data, 1 for validation data, and 1 for test data. The training data were augmented tenfold by randomly applying horizontal shift (range: 10% left/right), vertical shift (range: 10% up/down), brightness change (range: 0.3–1.2), and scaling (range: ± 10%). Evaluation indices of the constructed OCD classification model are accuracy, precision, recall, F1 score, and AUC. Accuracy and F1 score are defined by Eqs. (3) and (4). TN stands for true negative.

3 $$\mathrm{Accuracy}=\frac{\mathrm{TP}+\mathrm{TN}}{\mathrm{TP}+\mathrm{FP}+\mathrm{TN}+\mathrm{FN}}$$

4 $$\mathrm{F}1\mathrm{ score}=\frac{2\times \mathrm{Precision}\times \mathrm{Recall}}{\mathrm{Precision}+\mathrm{Recall}}$$

**Fig. 8:**
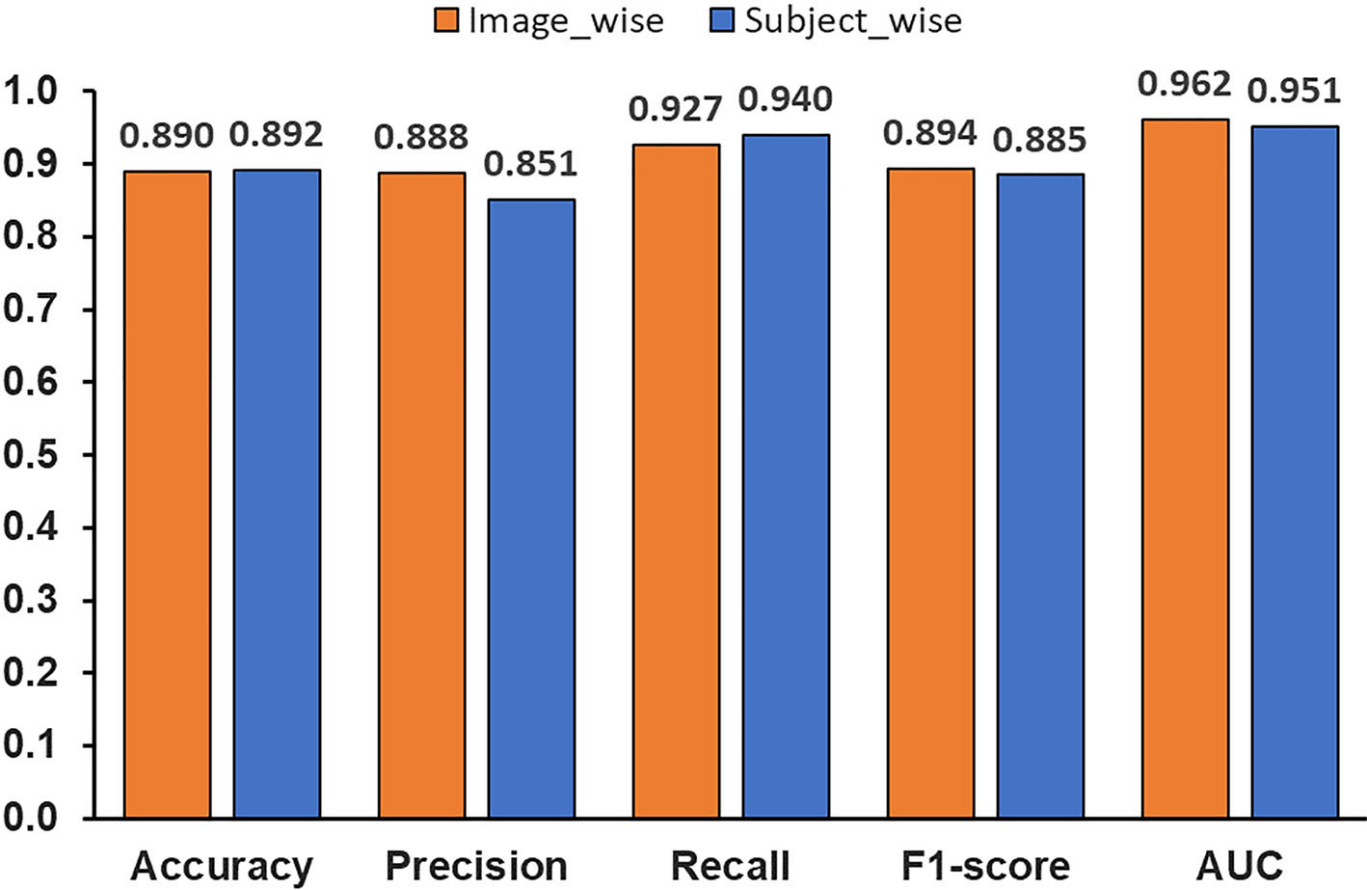
Average values of evaluation indices of OCD classification model in the proposed method

The average values for image-wise evaluation were 0.890 for accuracy, 0.888 for precision, 0.928 for recall, 0.885 for F1 score, and 0.952 for AUC in five-fold cross-validation. Since the number of images per subject varied, it is possible that many of the correct answers were given to subjects with multiple images in the per-image evaluation, and subjects with only one image were misclassified. Even in such a case, the result of the per-image evaluation may be good. In order to confirm such a possibility, a per subject evaluation (called subject-wise evaluation) is conducted. The subject-wise evaluation calculates the average probability of OCD for all images of one subject. Subjects with a mean probability of OCD exceeding 0.5 are assigned to the OCD class, and the others are assigned to the normal class. The average values of subject-wise evaluation in five-fold cross-validation were 0.892 for accuracy, 0.851 for precision, 0.940 for recall, 0.885 for F1 score, and 0.951 for AUC. It shows almost the same evaluation indices between image-wise and subject-wise, indicating that there is no risk of failing to detect OCD in certain subjects. Figure 8 shows the average values of the evaluation indices of five-fold cross-validation using the proposed method.

**Fig. 9:**
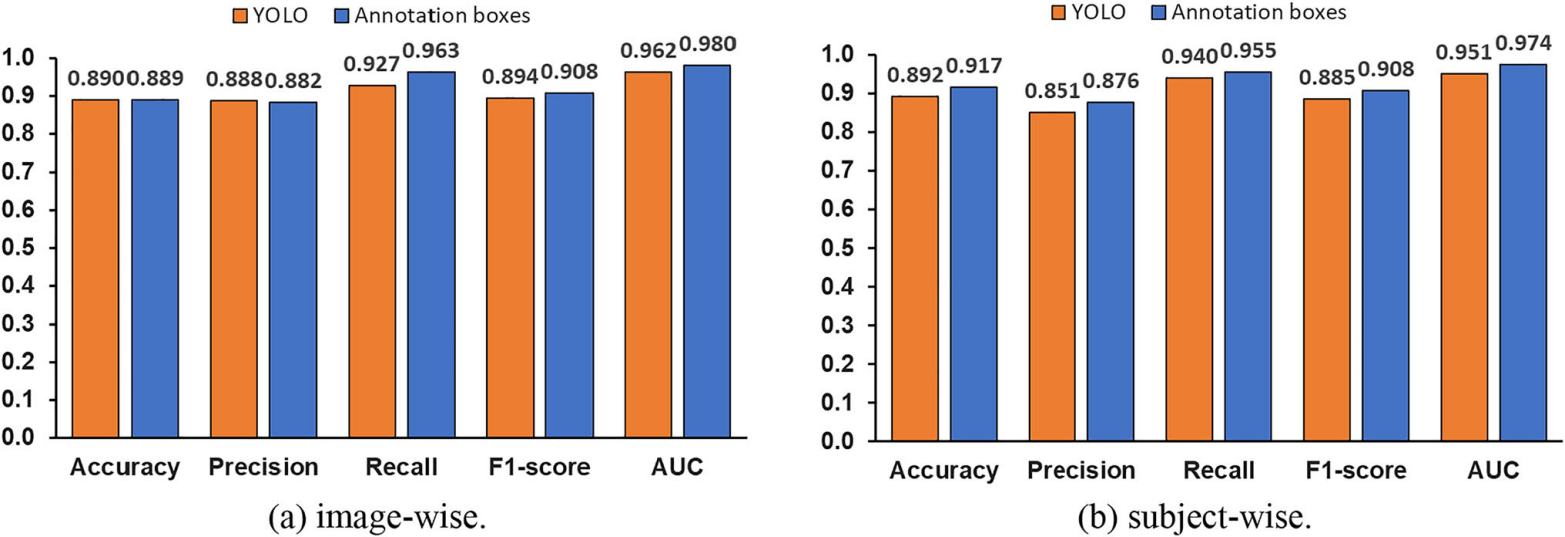
Effect of YOLO object detection on OCD classification accuracy

We also conducted an experiment to confirm whether the automated detection of the humeral capitellum by YOLO affects the accuracy of OCD classification. The experimental method is the same as before, and the cropped images by annotated bounding boxes are used to evaluate VGG16. Figure 9a compares the average values of the five-fold cross-validation between YOLO detected bounding boxes and annotated ones with image-wise evaluation, and Fig. 9b does the same with subject-wise evaluation.

**Fig. 10:**
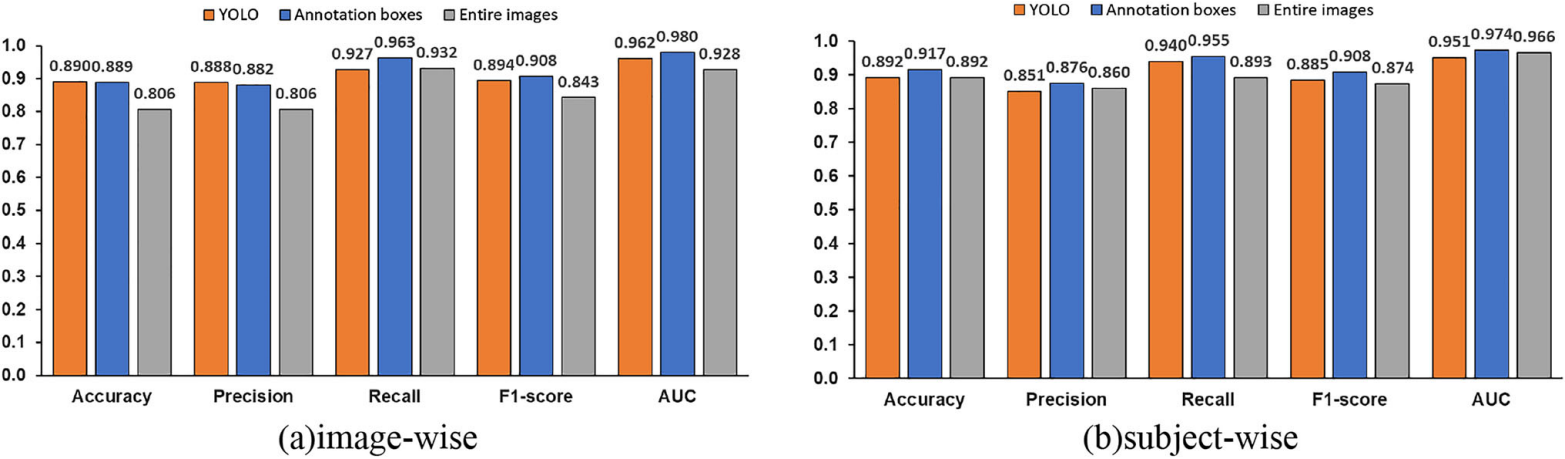
Comparison of evaluation indices for OCD classification with and without limitation of ROI

Next, we examined our hypothesis that ROI limitation improves the OCD classification performance. Another classification model was trained using the entire image. Experimental conditions were the same as before. The experimental results are shown in Fig. 10 in comparison with the previous method.

**Fig. 11:**
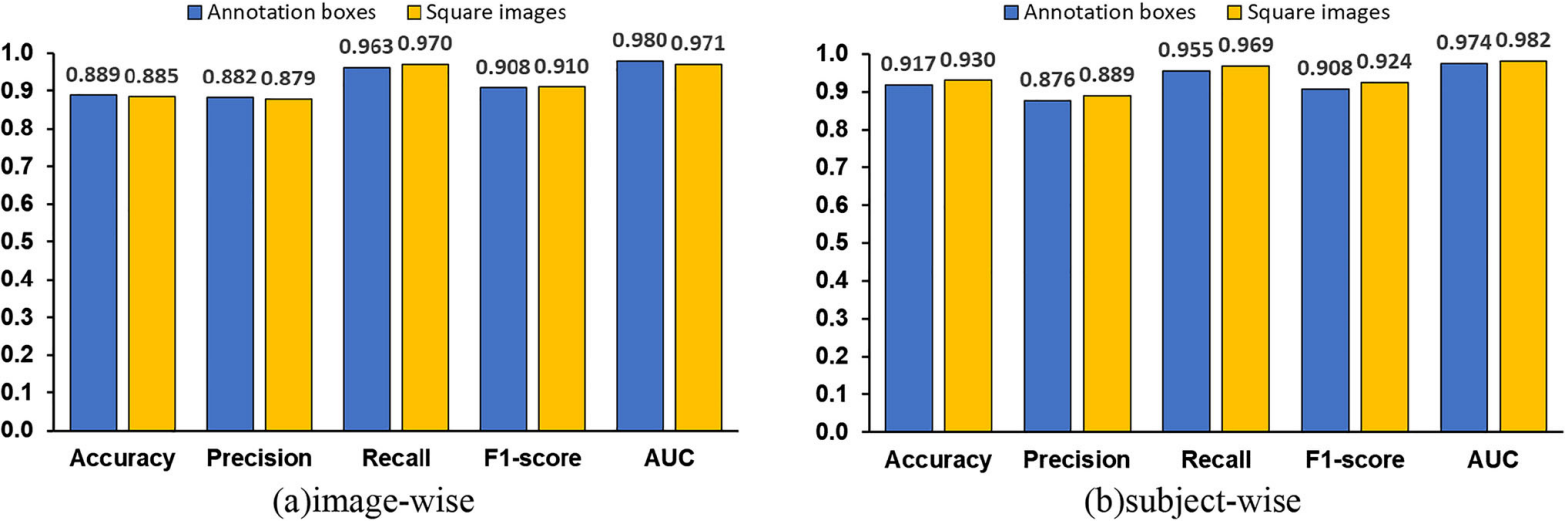
Differences in evaluation indices due to the different ways of carving out ROI.

The shape of the bounding box detected by YOLO varies from image to image. VGG16, which is used in the OCD classification model, has an input size of 224 × 224, which means that the image must be transformed into a square. Images that do not have the same aspect ratio may be horizontally or vertically elongated, which may affect the accuracy. To assess this effect, we manually cropped a square image along its longer direction. The manual operation was necessary to ensure an accurate comparison between the annotated boxes and the square ones. A classification model was constructed, and its performance was compared. The experimental conditions were the same as before, and the results are shown in Fig. 11.

## Discussion

### Effect of the humeral capitellum localization accuracy by YOLO on OCD classification

The proposed method detects the ROI using object detection with YOLOv5. However, the OCD classification model is constructed with the humeral capitellum image cropped from the annotated bounding boxes. As shown in Fig. 9, the accuracy for the bonding boxes detected by using YOLO was lower than that for the annotated bounding boxes. This suggests that the localization accuracy of YOLO has some influence on the accuracy of OCD classification. Therefore, improving the localization accuracy of the humeral capitellum by YOLO can increase the accuracy of OCD classification.

### Improvement of OCD classification accuracy by focusing on the humeral capitellum

We hypothesize that focusing the ROI on the humeral capitellum improves the OCD classification accuracy. To verify this hypothesis, we compared the model constructed using the entire image without ROI with the model that focuses on the humeral capitellum. The previous method by Shinohara et al. also detects the OCD from the entire image. Figure 10a and b shows the comparison of the results using the entire images, annotated bounding boxes, and bounding boxes detected by YOLO. Image-wise evaluation results in Fig. 10a indicate that all metrics using annotated bounding boxes are more accurate than those using the entire image. In Fig. 10b with subject-wise evaluation, all metrics using the annotated bounding boxes perform the best, and F1 score of YOLO detection is higher than the entire image, but AUC of YOLO detection is slightly lower. As mentioned earlier, the accuracy of YOLO’s object detection is not significantly affected by the accuracy of YOLO’s object localization, but the results are almost same as those of the model for the entire image, with a slight decrease in ACU due to the YOLO’s object localization performance. Results indicate that focusing ROI is an effective way to improve OCD classification accuracy. Improving the accuracy of YOLO’s humeral capitellum detection is a future issue, as seen from Fig. 9.

### Differences in accuracy among different ROI shapes

In this study, it was found that focusing the ROI on the humeral capitellum improved the accuracy compared the classification model using the entire image. As mentioned in the experiment section above, the ROI detected at step 1 varies in shape from image to image, and in many cases, the aspect ratio is not the same. Figure 11 compares the OCD classification performance between different ROI shapes in image-wise and subject-wise. Figure 11b shows that the accuracy improved slightly by square cropping.

## Conclusion

This study has proposed a method for detecting OCD from ultrasound images of the elbow. First, the humeral capitellum was detected by object detection using YOLOv5. We achieved a mAP70 of over 0.95 in five-fold cross-validation. The ROI was focused on the humeral capitellum by this object detection, and a classification model using VGG16 fine-tuning was constructed using the ROI images. As experimental results, we achieved an accuracy of 0.890, precision of 0.888, recall of 0.927, F1 score of 0.894, and AUC of 0.962 in image-wise evaluation. Furthermore, subject-wise evaluation shows an accuracy of 0.892, precision of 0.851, recall of 0.940, F1 score of 0.885, and AUC of 0.951. Our results concluded that focusing ROI on the humeral capitellum is an effective method for OCD classification from ultrasound images, since all evaluation indices were improved by using ROI.

A limitation of this study is that the performance of OCD classification is slightly degraded due to the localization performance of the humeral capitellum using YOLO. This can be improved by adjusting ROI location and shape to train VGG16. Another concern is that YOLO failed to detect the humeral capitellum in some frames of severe OCD cases. Although it does not significantly impair the effectiveness of the proposed method, it should be improved. And the results were obtained using the VGG16-based classification model, and future comparisons with other classification models are needed. As a future study, it is necessary to compare the OCD classification network model with other network structures to determine the optimal network structure. It is also necessary to compare the diagnostic performance of the model in this study with that of a medical specialist when the model is actually used for medical check-ups of OCD.
